# Supplementary material for: Landscape Connectivity Limits the Predicted Impact of Fungal Pathogen Invasion
Source: J Fungi (Basel). 2020 Oct 3;6(4):205. doi: 10.3390/jof6040205 (PMC7712934; doi:10.3390/jof6040205)
Supplement: Supplementary file 1 [file jof-06-00205-s001.pdf]

Supplementaty Table 1. Body temperature of six species of *Lyciasalamandra* measured in the field.

Date	species	coordinates	Status / point of measurement	Temperature (°C)	average temperature (°C)
1998/3/9	*L. flavimembris*	36°56'N, 28°16'E	active/ ground	14.2	10.6
1998/3/11	*L. b. billae*	36°58'N, 27°17'E	active/ ground	13.3	
2006/3/24	*L. l. luschani*	36°25'N, 29°14'E	active/ ground	12.6	
2006/3/25	*L. atifi*	37°11'N, 31°10'E	active/ ground	11.1	
2012/12/30	*L. l. basoglui*	36°22'N, 29°56'E	active/ air	7	
2012/12/31	*L. a. antalyana*	36°51'N, 30°35'E	active/ air	10	
2020/1/6	*L. b. arikani*	36°29'N, 30°25'E	active/ air	6	
1996/12/10	*L. b. billae*	36º47′N, 30º34′E	resting/ under stone	13.1	12.8
1996/12/10	*L. b. billae*	36°28'N, 30°20'E	resting/ under stone	13.7	
1996/12/12	*L. l. basoglui*	36°13'N, 29°45'E	resting/ under stone	8.4	
1998/3/10	*L. flavimembris*	36°28'N, 29°07'E	resting/ under stone	14.5	
1998/3/10	*L. l. luschani*	36°25'N, 29°14'E	resting/ under stone	16.4	
1998/3/10	*L. l. basoglui*	36°25'N, 29°14'E	resting/ under stone	12.6	
1998/3/10	*L. l. finikensis*	36°25'N, 29°14'E	resting/ under stone	14.1	
1998/3/11	*L. b. billae*	36°25'N, 29°14'E	resting/ under stone	13.1	
2004/2/27	*L. l. luschani*	36°47'N, 30°34'E	resting/ ground	10.5	
2004/2/27	*L. a. gocmeni*	37°0'N, 30°30'E	resting/ under stone	10.8	
2004/2/28	*L. atifi*	36°36'N, 32°2'E	resting/ ground	11	
2006/3/22	*L. b. billae*	36°24'N, 30°18'E	resting/ under stone	15.3	

Supplementaty Table 2. All (sub)species of *Lyciasalamandra* populations sampled for *B. salamandrivorans* presence, the last column indicates the Bayesian 95% credible intervals.

Species	Coordinates N	Coordinates E	Sample size	Prevalence	(Bayesian 95% credible interval)
L. atifi	36°48'N,	31°45'E	30	0.00	(0.00, 0.11)
L. antalyana	36°51'N,	30°35'E	30	0.00	(0.00, 0.11)
L. b. billae	36°47'N,	30°34'E	30	0.00	(0.00, 0.11)
L. b. irfani	36°42'N,	30°31'E	14	0.00	(0.00, 0.20)
L. b. yehudahi	36°32'N,	30°29'E	9	0.00	(0.00, 0.30)
L. b. yehudahi	36°36'N,	30°29'E	10	0.00	(0.00, 0.31)
L. b. yehudahi	36°35'N,	30°30'E	2	0.00	(0.00, 0.70)
L. b. yehudahi	total		21	0.00	(0.00, 0.16)
L. b. arikani	36°28'N,	30°25'E	10	0.00	(0.00, 0.31)
L. b. arikani	36°24'N,	30°25'E	22	0.00	(0.00, 0.15)
L. b. arikani	total		32	0.00	(0.00, 0.11)
L. billae	total		97	0.00	(0.00, 0.04)
L. l. finikensis	36°17'N,	30° 6'E	30	0.00	(0.00, 0.11)
L. l. basoglui	36°28'N,	29°24'E	30	0.00	(0.00, 0.11)
L. l. basoglui	36°12'N,	29°50'E	31	0.00	(0.00, 0.11)
L. l. basoglui	total		61	0.00	(0.00, 0.06)
L. l. luschani	36°22'N,	29°12'E	30	0.00	(0.00, 0.11)
L. luschani	total		121	0.00	(0.00, 0.03)
L. fazilae	36°46'N,	28°39'E	30	0.00	(0.00, 0.11)
L. flavimembris	36°55'N,	28°16'E	30	0.00	(0.00, 0.11)
L. helverseni	35°35'N	27°10'E	3	0.00	(0.00, 0.61)

L. helverseni	35°35'N	27°09'E	1		
L. helverseni	35°36'N	27°08'E	6	0.00	(0.00, 0.43)
L. helverseni	35°46'N	27°10'E	1		
L. helverseni	35°44'N	27°10'E	1		
L. helverseni	35°44'N	27°10'E	25	0.00	(0.00, 0.13)
L. helverseni	35°45'N	27°10'E	1		
L. helverseni	35°45'N	27°10'E	9	0.00	(0.00, 0.30)
L. helverseni	35°30'N	27°14'E	1		
L. helverseni	35°32'N	27°09'E	2	0.00	(0.00, 0.70)
L. helverseni	35°33'N	27°08'E	5	0.00	(0.00, 0.44)
L. helverseni	35°32'N	27°08'E	2	0.00	(0.00, 0.70)
L. helverseni	35°32'N	27°08'E	1		
L. helverseni	35°32'N	27°08'E	1		
L. helverseni	35°29'N	27°10'E	3	0.00	(0.00, 0.61)
L. helverseni	35°29'N	27°09'E	5	0.00	(0.00, 0.44)
L. helverseni	35°36'N	27°08'E	2	0.00	(0.00, 0.70)
L. helverseni	35°44'N	27°11'E	1		
L. helverseni	35°44'N	27°10'E	4	0.00	(0.00, 0.52)
L. helverseni	N/A	N/A	2	0.00	(0.00, 0.70)
L. helverseni	35°34'N	27°08'E	5	0.00	(0.00, 0.44)
L. helverseni	35°33'N	27°08'E	1		
L. helverseni	35°35'N	27°09'E	4	0.00	(0.00, 0.52)
L. helverseni	35°37'N	27°08'E	4	0.00	(0.00, 0.52)
L. helverseni	35°35'N	27°09'E	3	0.00	(0.00, 0.61)
L. helverseni	35°23'N	26°56'E	2	0.00	(0.00, 0.70)
L. helverseni	35°24'N	26°58'E	7	0.00	(0.00, 0.36)

L. helverseni	35°24'N	26°57'E	39	0.00	(0.00, 0.09)
L. helverseni	35°23'N	26°56'E	15	0.00	(0.00, 0.19)
L. helverseni	total		156	0.00	(0.00, 0.03)
Lyciasalamandra	**total**		**494**	**0.00**	**(0.00, 0.01)**

Supplementaty Table 3. Recorded temperature of 6 *Lyciasalamandra* species from 1997 to 1999.

							Temperatures				T > 25°C		T not < 25°C	
**Species**	**Latitude**	**Longitude**	**Altitude a.s.l. (m)**	**Slope**	**Logger place**	**Measure-ment period**	**Measure-ments**	**Min (°C)**	**Mean (°C)**	**Max (°C)**	**Period**	**n**	**n**	**Max. n consecutive days**
1997/1998														
L. atifi	36°81'N	31°77'E	610	SW	2 m above ground	23.02.1997-21.02.1998	2901	-2.2	15.8	39.8	22.04.1997 - 17.10.1997	145	3	1
L. atifi	36°81'N	31°77'E	610	SW	under stone	23.02.1997-21.02.1998	2901	5.0	15.9	29.2	19.06.1997 - 19.09.1997	88	18	7
L. atifi	36°81'N	31°77'E	610	SW	ca. 30 cm inside stone wall	23.02.1997-21.02.1998	2901	3.3	16.2	29.2	21.06.1997 - 19.09.1997	83	69	42
L. atifi	36°81'N	31°77'E	610	SW	ca. 30 cm inside stone wall	23.02.1997-21.02.1998	2901	3.5	16.7	31.6	20.06.1997 - 24.09.1997	96	87	44
L. billae	36°79'N	30°77'E	30	SE	2 m above ground	20.02.1997-20.02.1998	2925	4.0	18.0	38	12.05.1997 - 17.10.1997	119	13	5
L. billae	36°79'N	30°77'E	30	SE	under	20.02.1997-	2925	8.4	18.4	31.4	19.06.1997	91	82	44

					stone	20.02.1998					- 19.09.1997			
L. billae	36°79'N	30°77'E	30	SE	ca. 30 cm in leaf litter	20.02.1997-20.02.1998	2925	8.4	20.4	33.5	16.06.1997 - 17.10.1997	114	92	84
L. billae	36°79'N	30°77'E	30	SE	ca. 20 cm in mouse hole	20.02.1997-20.02.1998	2925	6.6	18.0	31.4	30.06.1997 - 20.09.1997	85	77	53
1998/1999														
L. atifi	36°81'N	31°77'E	610	SW	under stone	26.02.1998-20.02.1999	2870	5.4	17.7	29.6	01.07.1998 - 16.09.1999	72	57	50
L. atifi	36°59'N	32°26'E	750	NE	under stone	21.02.1998-24.02.1999	2956	3.1	13.4	26.3	04.08.1998 - 11.08.1998	7	0	0
L. atifi	36°64'N	32°57'E	1110	NE	under stone	21.02.1998-24.02.1999	2955	0.7	11.5	25.2	08.08.1998	1	0	0
L. atifi	37°20'N	31°17'E	570	SW	under stone	24.02.1998-20.02.1999	2887	2.7	16.0	31.4	17.06.1998 - 15.09.1999	88	61	54
L. antalyana	36°85'N	30°59'E	125	SE	under stone	25.02.1998-19.02.1999	2870	7.3	22.1	42.4	16.04.1998 - 24.10.1999	152	125	123
L. antalyana	37°12'N	30°53'E	655	NE	under	25.02.1998-	2848	1.1	14.8	32.6	17.06.1998	88	18	14

					stone	17.02.1999					- 16.09.1999			
L. billae	36°79'N	30°57'E	30	SE	under stone	20.02.1998-19.02.1999	2910	9.1	19.8	32.6	24.06.1998 - 10.10.1999	99	86	73
L. billae	36°79'N	30°57'E	30	NW	ca. 70 cm inside boulder field	27.02.1998-19.02.1999	2857	10.7	19.0	29.6	28.06.1998 - 22.09.1999	87	87	86
L. luschani	36°51'N	30°62'E	555	SW	under stone	01.03.1998-12.02.1999	2784	1.9	18.9	35.3	10.06.1998 - 24.10.1999	165	64	23
L. luschani	36°28'N	30°15'E	40	S	under stone	10.03.1998-12.02.1999	2712	8.5	22.5	37.8	11.04.1998 - 30.10.1999	168	97	91
L. luschani	36°19'N	29°88'E	30	NW	under stone	02.03.1998-13.02.1999	2784	8.0	20.0	33.3	07.06.1998 - 20.09.1999	75	94	87
L. luschani	36°39'N	29°71'E	840	S	under stone	01.03.1998-12.02.1999	2784	4.3	17.5	33.0	13.06.1998 - 28.09.1999	101	75	67
L. luschani	36°43'N	29°19'E	365	N	under stone	04.03.1998-13.02.1999	2769	3.5	16.5	31.1	12.06.1998 - 10.09.1999	85	33	25
L. fazilae	36°78'N	28°66'E	100	N	under	06.03.1998-	2768	5.3	19.0	36.0	31.05.1998	11	86	81

					stone	15.02.1999					- 20.09.1999	3		
L. fazilae	36°80'N	29°19'E	1025	NE	under stone	05.03.1998- 14.02.1999	2765	-0.1	11.8	23.0	none	0	0	0
L. flavimembris	36°81'N	28°30'E	115	E	under stone	07.03.1998- 15.02.1999	2761	3.7	19.5	41.3	01.05.1998 - 28.09.1999	13 6	54	34

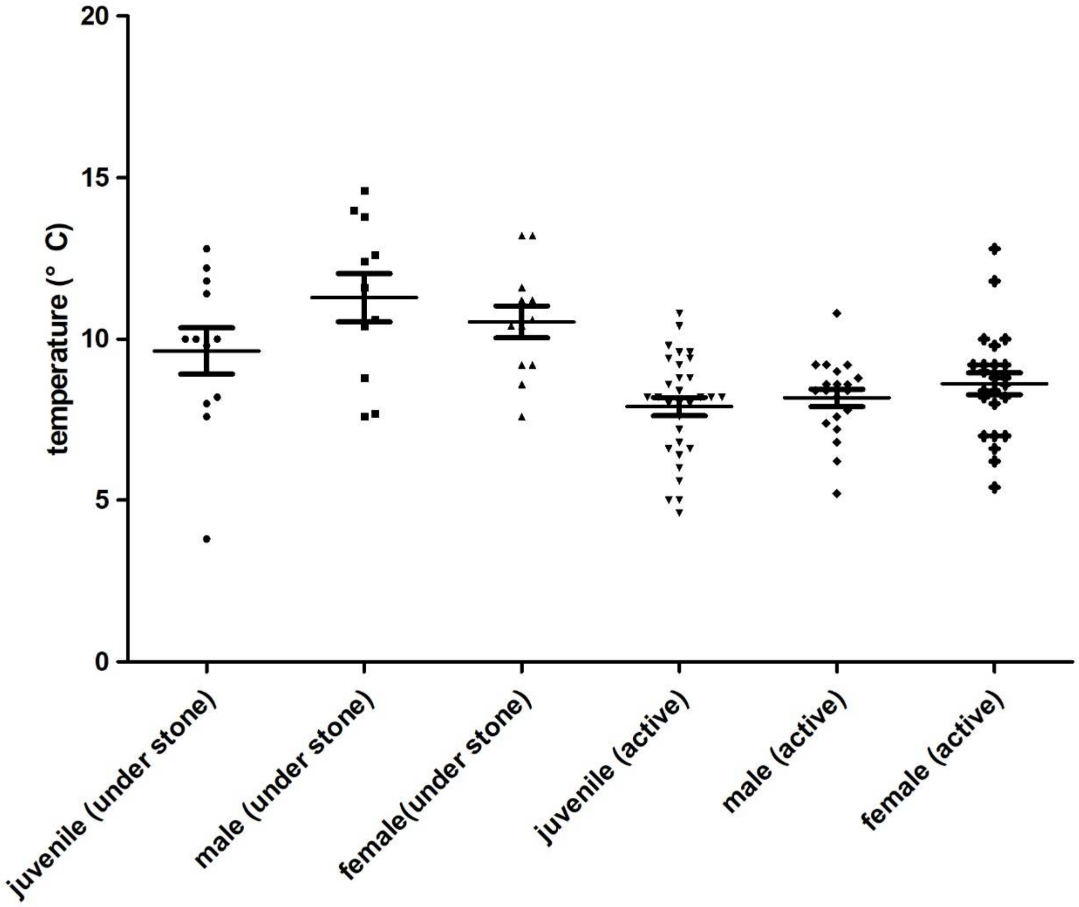

Supplementaty Figure 1. Dot plot of the body temperature of *L. helverseni* in °C measured in the field.
